# Efeito Anti-Hipertensivo de Novos Agonistas do Receptor de Adenosina em Ratos Espontaneamente Hipertensos

**DOI:** 10.36660/abc.20230405

**Published:** 2024-03-15

**Authors:** Bruna de Souza Rocha, Jaqueline Soares da Silva, Júlia Galvez Bulhões Pedreira, Tadeu Lima Montagnoli, Eliezer Jesus Barreiro, Gisele Zapata-Sudo

**Affiliations:** 1 Universidade Federal do Rio de Janeiro Rio de Janeiro RJ Brasil Universidade Federal do Rio de Janeiro, Rio de Janeiro, RJ – Brasil

**Keywords:** Hipertensão, Vasodilatação, Canais de Cálcio, Receptores Purinérgicos P1

## Abstract

**Fundamento:**

A hipertensão arterial sistêmica é um fator de risco para disfunções cardíacas, renais e metabólicas. A busca por novas estratégias para prevenir e tratar doenças cardiovasculares levou à síntese de novas ***N*** -acilidrazonas para produzir efeito anti-hipertensivo. Os receptores de adenosina são um alvo alternativo para reduzir a pressão arterial devido à sua ação vasodilatadora e propriedades antioxidantes, que podem reduzir o estresse oxidativo característico da hipertensão arterial sistêmica.

**Objetivo:**

Avaliar o perfil anti-hipertensivo de novos compostos contendo selênio desenvolvidos para melhorar sua interação com os receptores de adenosina.

**Métodos:**

Foi avaliada a reatividade vascular, registrando-se a tensão isométrica da aorta torácica pré-contraída de ratos Wistar machos após exposição a concentrações crescentes de cada derivado (0,1 a 100 μM). Para investigar o efeito anti-hipertensivo em ratos espontaneamente hipertensos, foram determinadas a pressão arterial sistólica, pressão arterial diastólica, pressão arterial média e a frequência cardíaca após administração intravenosa de 10 e 30 μmol/kg do composto selecionado LASSBio-2062.

**Resultados:**

Os compostos denominados LASSBio-2062, LASSBio-2063, LASSBio-2075, LASSBio-2076, LASSBio-2084, LASSBio-430, LASSBio-2092 e LASSBio-2093 promoveram vasodilatação com concentrações efetivas médias de 15,5 ± 6,5; 14,6 ± 2,9; 18,7 ± 9,6; 6,7 ± 4,1; > 100; 6,0 ± 3,6; 37,8 ± 11,8; e 15,9 ± 5,7 μM, respectivamente. O LASSBio-2062 (30 μmol/kg) reduziu a pressão arterial média em ratos espontaneamente hipertensos de 124,6 ± 8,6 para 72,0 ± 12,3 mmHg (p < 0,05). A ativação do receptor de adenosina subtipo A_3_ e dos canais de potássio parece estar envolvida no efeito anti-hipertensivo do LASSBio-2062.

**Conclusões:**

O novo agonista do receptor de adenosina e ativador dos canais de potássio é um potencial agente terapêutico para o tratamento da hipertensão arterial sistêmica.

**Figure:**
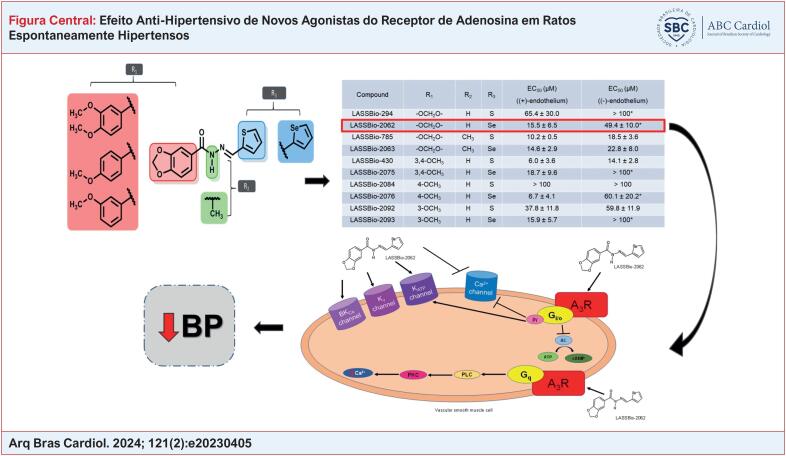

## Introdução

Segundo estimativas da Organização Mundial da Saúde, a morte de aproximadamente 23,6 milhões de pessoas ocorrerá até 2030 em decorrência de doenças cardiovasculares.^1^ Mesmo com a grande variedade de tratamentos existentes, a hipertensão arterial continua a ser a principal causa de morte cardíaca em todo o mundo, sendo responsável por 10,4 milhões de mortes por ano, com o maior número de indivíduos afetados pela hipertensão em países de baixa e média renda.^2 - 5^

Embora existam diversas terapias modernas para o tratamento da hipertensão, ela continua sendo a principal causa de doenças cardiovasculares, como cardiopatia isquêmica, insuficiência cardíaca e acidente vascular cerebral, agravadas pelo envelhecimento populacional.^6 - 9^

Devido ao caráter assintomático, a hipertensão arterial sistêmica pode evoluir com alterações estruturais e/ou funcionais em órgãos-alvo (coração, cérebro, rins e vasos).^10^ É importante buscar novas estratégias para a prevenção e o tratamento da hipertensão; dessa maneira, foram projetadas novas *N* -acilidrazonas, sintetizadas a partir da 3,4-metilenodioxibenzoil-2-tienilhidrazona (LASSBio-294) para testar a atividade vasodilatadora. O LASSBio-294, caracterizado como agente inotrópico cardíaco positivo com atividade vasodilatadora, preveniu a disfunção cardíaca induzida por infarto do miocárdio em ratos normotensos e hipertensos, possivelmente devido à ativação de receptores de adenosina A_2A_.^11 - 16^ Para melhorar a interação entre a molécula e seu sítio de ação, os novos compostos possuem o átomo de enxofre (–S) em vez de selênio (–Se), o que confere uma conformação restrita, levando a uma melhor interação composto-receptor^17^ e, consequentemente, aumentando a potência vasodilatadora. Algumas alterações na estrutura molecular poderiam gerar compostos com mecanismos de ação multialvos, favorecendo a adesão dos pacientes à terapia, uma vez que a polifarmácia é comum para o controle da hipertensão arterial na maioria dos pacientes.^18^ A busca por novas terapias para hipertensão é uma tentativa de aumentar a adesão dos pacientes ao tratamento, pois este é um grande desafio para os cardiologistas, devido ao uso de múltiplos medicamentos e/ou administrações, alta incidência de efeitos adversos e doença primária não diagnosticada.^19 , 20^

O principal objetivo deste estudo foi identificar uma nova N-acilidrazona, na qual a substituição do átomo de enxofre pelo selênio pudesse aumentar a atividade vasodilatadora e produzir um efeito anti-hipertensivo. Com base nos resultados, foi selecionado o LASSBio-2062, que foi um vasodilatador potente através da ativação dos receptores de adenosina A_3_ e dos canais K. O LASSBio-2062 poderia ser uma nova abordagem à monoterapia para hipertensão, uma vez que promoveu a normalização da pressão arterial em ratos espontaneamente hipertensos (SHR).

## Métodos

Os experimentos estavam de acordo com o Comitê de Ética no Uso de Animais da Universidade Federal do Rio de Janeiro (017/19). Os animais incluídos neste estudo foram divididos aleatoriamente, com base no peso e/ou idade. Cinco ratos Wistar machos (220 a 250 g) ou quatro SHR machos (12 a 15 semanas e 220 a 250 g) foram utilizados para cada protocolo conduzido em experimentos *in vitro* e *in vivo* , respectivamente. Os SHR foram tratados com injeção intravenosa de dose única de LASSBio-2062 e foram eutanasiados com administração intraperitoneal (IP) de tiopental (120 mg/kg) imediatamente após a medição da pressão arterial.^21^ Todos os animais foram mantidos sob controle de temperatura e ciclo claro/escuro de 12 horas, com acesso a água e alimentos *ad libitum.*

### Compostos

**Figura 1:**
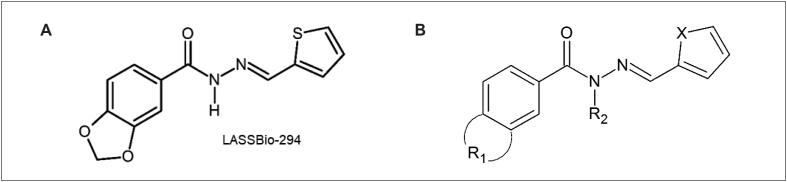
(A) Estrutura química do LASSBio-294. (B) Estrutura indicando os pontos de mudança na molécula que originou os seguintes análogos: X = S ou Se; R1 = -OCH_2_O- ou -3,4-OCH_3_ ou -3-OCH_3_ ou -4-OCH_3_ e R2 = -H ou -CH_3_.

Os derivados ( Figura 1 ) foram sintetizados e fornecidos pelo Laboratório de Avaliação e Síntese de Substâncias Bioativas (LASSBio^®^) da Universidade Federal do Rio de Janeiro. O protótipo, 3,4-metilenodioxibenzoil-2-tienilhidrazona e seus análogos foram denominados LASSBio-294, LASSBio-2062, LASSBio-2063, LASSBio-430, LASSBio-2075, LASSBio-2084, LASSBio-2076, LASSBio-2092 e LASSBio-2093.

### Experimentos *in vitro*

#### Reatividade vascular

A aorta torácica de ratos Wistar machos foi removida e cortada em anéis de 2 a 3 mm, os quais foram posicionados em câmaras experimentais contendo 20 mL de solução de Tyrode modificada composta por (em mM): 123 NaCl; 4,7 KCl; 15,5 NaHCO_3_; 1,2 CaCl_2_; 1,2 KH_2_PO_4_; 1,2 MgCl_2_; e 11,5 glicose. Foram oxigenados com mistura de carbogênio a 37 °C e pH 7,4. Após 2 horas de equilíbrio e sob 1 g de tensão inicial, os anéis aórticos foram inicialmente expostos a 10 μM de fenilefrina para promover a contração máxima registrada por meio de um transdutor de força (MLT884, ADInstruments, Austrália), conectado a um sistema de aquisição (Powerlab, ADInstruments, Austrália), utilizando o programa Lab Chart (versão 7.0, ADInstruments, Austrália). Os anéis pré-contraídos foram expostos a 10 μM de acetilcolina para promover relaxamento que, quando igual ou superior a 80%, determinou a presença de endotélio intacto. Em contrapartida, quando os anéis aórticos submetidos à remoção mecânica do endotélio produziram menos de 10% de relaxamento, houve falta de endotélio funcional. Concentrações crescentes (0,1 a 100 μM) de todos os análogos (LASSBio-2062, LASSBio-2063, LASSBio-430, LASSBio-2075, LASSBio-2084, LASSBio-2076, LASSBio-2092, LASSBio-2093) foram adicionadas às câmaras contendo os anéis aórticos contraídos com fenilefrina para obter as curvas de concentração-resposta de relaxamento.^22^

Investigamos os mecanismos envolvidos no relaxamento vascular induzido pelo composto selecionado, LASSBio-2062, com pré-exposição de anéis aórticos com endotélio intacto a um antagonista do receptor de adenosina A_2A_, ZM-241385 (0,1 μM). Anéis aórticos sem endotélio foram pré-incubados com um dos seguintes: um antagonista do receptor de adenosina A_3_ (MRE 3008F20, 0,1 μM); um antagonista dos canais de potássio sensível a ATP (glibenclamida, 10 μM); um bloqueador do canal de potássio voltagem-dependente (4-aminopiridina, 3 mM); ou um bloqueador do canal de potássio cálcio-dependente de alta condutância (cloreto de tetraetilamônio, 3 mM).

### Experimentos *in vivo*

#### Medição da pressão arterial

Os SHR sob anestesia com cetamina (80 mg/kg, IP) e xilazina (15 mg/kg, IP) foram submetidos ao cateterismo, utilizando um cateter preenchido com solução salina e heparina, conectado a um transdutor de pressão (MLT884, ADInstruments, Austrália). Esse procedimento possibilitou o registro das pressões arteriais sistólica e diastólica e da frequência cardíaca durante a injeção intravenosa de LASSBio-2062 (10 e 30 μmol/kg) pela veia jugular esquerda. Os parâmetros foram obtidos utilizando um sistema de aquisição (Powerlab, ADInstruments, Austrália) e o software Lab Chart (versão 7.0, ADInstruments, Austrália).

### Análise estatística

Os dados são expressos como média ± erro padrão da média. Para cada substância testada, calculamos a concentração que causou metade do efeito máximo de relaxamento (EC_50_). Foi utilizada a análise ANOVA bidirecional seguida de pós-teste de Tukey para avaliar dados de experimentos *in vitro* . Foi realizada a análise pelo teste t de Student em dados de experimentos *in vivo* . A diferença entre os grupos experimentais foi considerada estatisticamente significativa quando p < 0,05. Foram considerados poder estatístico (β) de 80% e taxa de falsos positivos (α) de 5%. Para identificar alterações com poder estatístico (β) de 80% e taxa de falsos positivos (α) de 5%, foi necessário um número experimental de 5 animais para o protocolo *in vitro* . Dessa maneira, o teste estatístico diferencia as respostas com variação de pelo menos 15% e desvio padrão médio de 8,0%, em percentuais relativos à média do grupo controle. Quanto aos protocolos *in vivo* , o desvio padrão médio foi de 7,0% em relação ao grupo controle, com número experimental de 4 animais para cada grupo. Foi utilizado o software http://powerandsamplesize.com/Calculators para calcular o tamanho da amostra.

## Resultados

### Reatividade vascular em anéis aórticos com ou sem endotélio intacto

**Figura 2:**
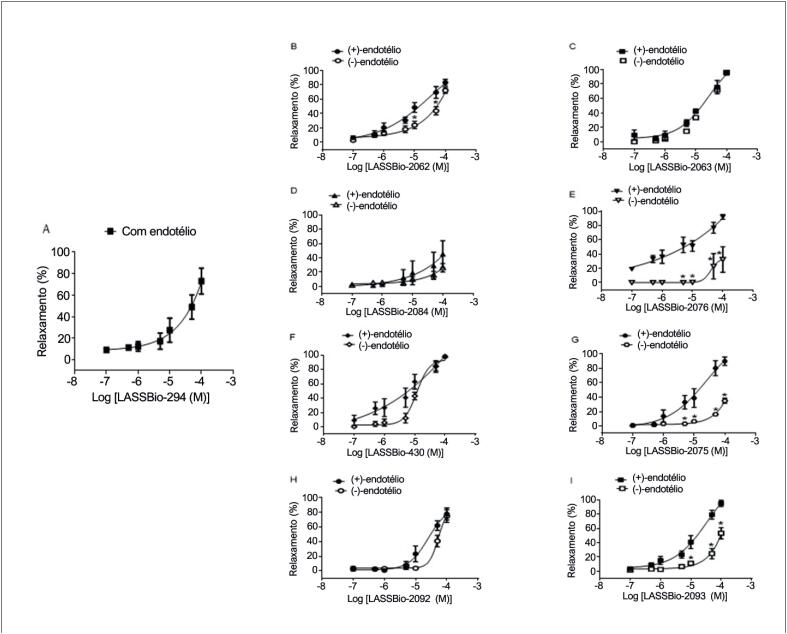
Curvas de concentração-resposta vascular para (A) LASSBio-294; (B) LASSBio-2062; (C) LASSBio-2063; (D) LASSBio-2084; (E) LASSBio-2076;(F) LASSBio-430; (G) LASSBio-2075; (H) LASSBio-2092; e (I) LASSBio-2093 em anéis aórticos com ou sem endotélio funcional. Os dados são apresentados como média ± erro padrão da média. *p < 0,05 comparado ao endotélio intacto. Análise por ANOVA bidirecional seguida de pós-teste de Tukey.

As *N* -acilidrazonas foram avaliadas quanto ao potencial de produzir vasodilatação em comparação ao protótipo (LASSBio-294). Cada derivado foi testado em anéis aórticos com e sem endotélio intacto, que foram expostos a concentrações crescentes (0,1 a 100 μM). A Tabela 1 inclui os valores de EC_50_ nos anéis aórticos. As curvas de concentração-relaxamento vascular para todos os análogos testados são exibidas na Figura 2. A remoção mecânica do endotélio aumentou a EC_50_ de 15,5 ± 6,5 para 49,4 ± 10,0 μM para o LASSBio-2062, indicando que o seu efeito é parcialmente dependente da integridade funcional do endotélio vascular ( Figura 2A ). Os valores de EC_50_ para o LASSBio-2063 foram 14,6 ± 2,9 e 22,8 ± 8,0 μM em anéis com e sem endotélio, respectivamente, sugerindo que não depende da integridade endotelial ( Figura 2B ).

**Tabela 1:** EC_50_ de análogos para relaxamento vascular em anéis aórticos de ratos

Composto	R_1_	R_2_	X	EC_50_ (µM) (com endotélio)	EC_50_ (µM) (sem endotélio)
LASSBio-294	-OCH_2_O-	H	S	65,4 ± 30,0	> 100 *
LASSBio-2062	-OCH_2_O-	H	Se	15,5 ± 6,5	49,4 ± 10,0 *
LASSBio-785	-OCH_2_O-	CH_3_	S	10,2 ± 0,5	18,5 ± 3,6
LASSBio-2063	-OCH_2_O-	CH_3_	Se	14,6 ± 2,9	22,8 ± 8,0
LASSBio-430	3,4-OCH_3_	H	S	6,0 ± 3,6	14,1 ± 2,8
LASSBio-2075	3,4-OCH_3_	H	Se	18,7 ± 9,6	> 100 *
LASSBio-2084	4-OCH_3_	H	S	> 100	> 100
LASSBio-2076	4-OCH_3_	H	Se	6,7 ± 4,1	60,1 ± 20,2 *
LASSBio-2092	3-OCH_3_	H	S	37,8 ± 11,8	59,8 ± 11,9
LASSBio-2093	3-OCH_3_	H	Se	15,9 ± 5,7	> 100 *

* *p < 0,05, em comparação com os dados com endotélio intacto usando análise ANOVA bidirecional. EC_50_: concentração que produziu metade do efeito máximo; R_1_: radical 1; R_2_: radical 2.*

O *design* e a síntese do LASSBio-430 basearam-se na abertura do anel 1,3-benzodioxol, cujos resultados indicam que essa alteração estrutural pode levar ao aumento da eficácia e da potência da vasodilatação, com relaxamento máximo independente do endotélio de 99,2% ± 0,8 % e EC_50_ de 6,0 ± 3,6 μM.

Em contrapartida, o LASSBio-2075, que possui presença adicional do anel selenofênico no lugar do anel tiofênico, possui EC_50_ de 18,7 ± 9,6 e o relaxamento vascular é dependente da integridade do endotélio, porque o valor passou a ser > 100 μM quando o endotélio foi removido. O LASSBio-2084 não apresentou melhora na potência indicando que a metoxilação não interfere na reatividade vascular. Da mesma forma, o relaxamento vascular induzido pelo LASSBio-2093 foi dependente da integridade do endotélio, uma vez que a EC_50_ aumentou de 15,9 ± 5,7 para > 100 μM.

A dependência da integridade vascular para a ação vasodilatadora do LASSBio-2076 foi confirmada pelo aumento da EC_50_ de 6,7 ± 4,1 para 60,1 ± 20,2 μM. Assim, a metoxilação somada à presença do anel selenofênico promoveu um aumento na potência do LASSBio-2076 em anéis aórticos com endotélio íntegro. O análogo LASSBio-2092 apresentou valores de EC_50_ de 37,8 ± 11,8 e 59,8 ± 11,9 μM em anéis aórticos com e sem endotélio, respectivamente.

O LASSBio-2062 foi selecionado para investigação dos mecanismos envolvidos no relaxamento vascular, devido à grande semelhança estrutural com o composto original.

### Investigação dos mecanismos envolvidos na ação vasodilatadora do LASSBio-2062

#### Via do receptor de adenosina

**Figura 3:**
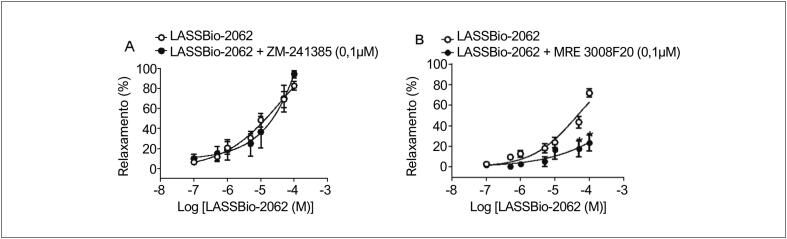
Curvas concentração-relaxamento vascular do LASSBio-2062 em anéis aórticos com endotélio: (A) na ausência ou presença de ZM-241385 (0,1 μM), antagonista do receptor A2A da adenosina; e (B) na presença de MRE 3008F20 (0,1 μM), um antagonista do receptor A3 de adenosina. Os dados são apresentados como média ± erro padrão da média. *p < 0,05 comparado ao endotélio intacto. Análise por ANOVA dois fatores seguida de pós-teste de Tukey.

Não houve alteração no relaxamento vascular promovido pelo LASSBio-2062 na presença de um antagonista do receptor de adenosina A_2A_, ZM-241385 ( Figura 3A ). Porém, quando os anéis aórticos foram pré-incubados com o antagonista do receptor de adenosina A_3_, MRE 3008F20, a curva de concentração-resposta apresentou desvio para a direita ( Figura 3B ). Além disso, o relaxamento máximo induzido pelo LASSBio-2062 alterou de 72,1% ± 4,0% para 23,6% ± 7,7% na presença do MRE 3008F20. Esses dados indicam que o composto promove vasodilatação pela ativação do receptor de adenosina tipo A_3_, mas não do receptor de adenosina tipo A_2A_.

#### Envolvimento de canais de potássio

**Figura 4:**
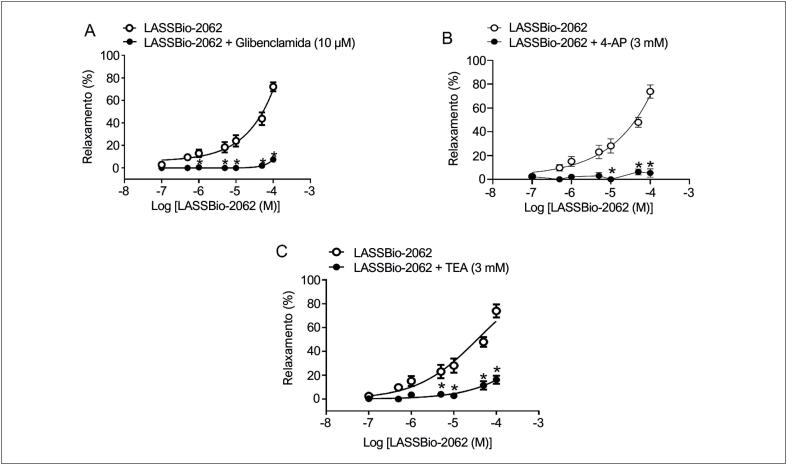
Curvas de concentração-relaxamento vascular do LASSBio-2062 em anéis aórticos sem endotélio na ausência ou presença de: (A) glibenclamida (10 μM), um bloqueador do canal de potássio sensível a ATP; (B) 4-aminopiridina (4-AP, 3 mM), um bloqueador do canal de potássio voltagem- dependente; e (C) cloreto de tetraetilamônio (TEA, 3 mM), um bloqueador do canal de potássio cálcio-dependente de alta condutância. Os dados são expressos como média ± erro padrão da média. *p < 0,05 comparado ao controle. Análise por ANOVA bidirecional seguida de pós-teste de Tukey.

Quando os anéis aórticos foram pré-incubados com glibenclamida (10 μM), que é um bloqueador do canal de potássio sensível a ATP, a resposta de concentrações crescentes de LASSBio-2062 foi alterada ( Figura 4A ). Quando na presença de 4-aminopiridina ( Figura 4B ) ou cloreto de tetraetilamônio ( Figura 4C ), a curva concentração-relaxamento do LASSio-2062 se deslocou para a direita, indicando que a vasodilatação induzida pelo LASSBio-2062 pode ser consequente à ativação do canal de potássio sensível a ATP, do canal de potássio voltagem-dependente e do canal de potássio cálcio-dependente ( Figura 4 ).

#### Influência do influxo de cálcio na ação das *N* -acilidrazonas

**Figura 5:**
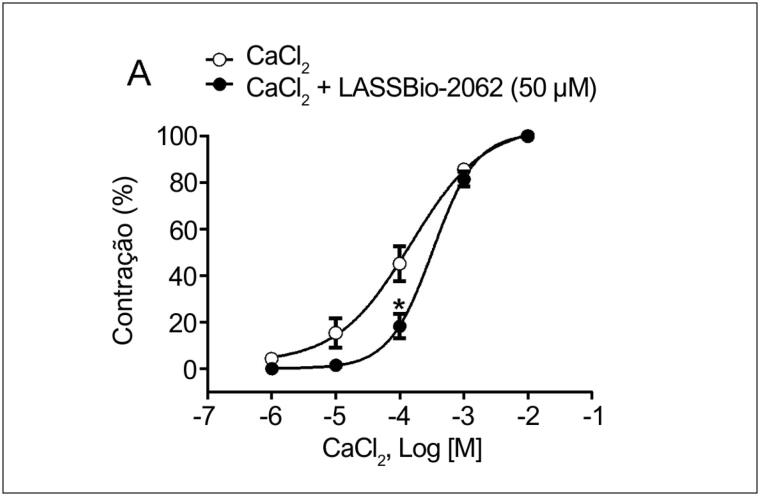
Curvas de concentração-resposta de CaCl_2_ na ausência ou presença do LASSBio-2062 em anéis aórticos sem endotélio. Os dados são expressos como média ± erro padrão da média. *p < 0,05 comparado ao controle. Análise por ANOVA bidirecional seguida de pós-teste de Tukey.

Para verificar se o LASSBio-2062 induzia vasodilatação através de uma interferência adicional na concentração intracelular de Ca^2+^, anéis aórticos sem endotélio foram expostos a concentrações crescentes de CaCl_2_ (1 a 1000 µM), na ausência ou presença do análogo (50 μM) ( Figura 5 ). A curva concentração-resposta se deslocou para a direita com a exposição ao LASSBio-2062, com aumento da EC_50_ de 157,6 ± 51,3 para 420,0 ± 11,0 μM (p < 0,05).

#### Efeito anti-hipertensivo do LASSBio-2062

**Figura 6:**
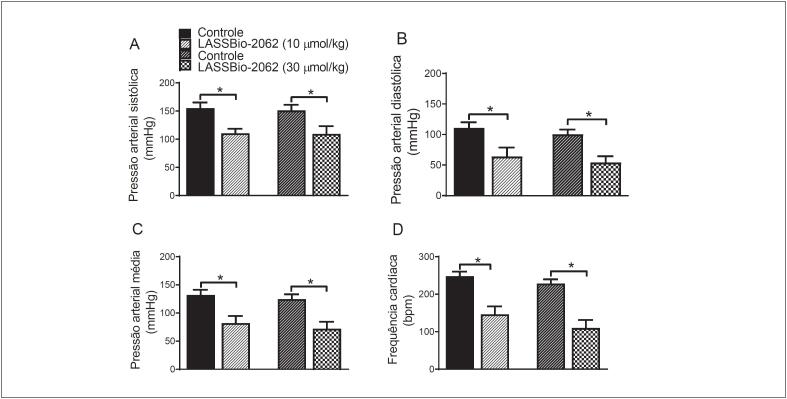
Parâmetros hemodinâmicos observados em ratos espontaneamente hipertensos antes (controle) e após administração intravenosa de LASSBio-2062. (A) Pressão sistólica; (B) pressão diastólica; (C) pressão média; e (D) frequência cardíaca, medidas antes e após a administração de 10 μmol/kg (n = 4) e 30 μmol/kg (n = 4) de LASSBio-2062. Os dados são expressos como média ± erro padrão da média. *p < 0,05 versus controle. Análise pelo teste t de Student.

Após administração intravenosa do LASSBio-2062, a pressão sistólica dos SHR reduziu de 155,1 ± 10,1 para 110,6 ± 8,0 e de 151,1 ± 10,0 para 109,4 ± 13,8 mmHg nas doses de 10 e 30 μmol/kg, respectivamente. A pressão diastólica também foi reduzida com a administração de LASSBio-2062 de 111,0 ± 9,1 para 63,9 ± 14,7 (10 μmol/kg) e de 100,2 ± 7,8 para 54,0 ± 10,4 mmHg (30 μmol/kg). A pressão arterial média diminuiu de 132,0 ± 9,2 e 124,6 ± 8,6 mmHg (grupo controle) para 82,1 ± 12,6 (p < 0,05) e 72,0 ± 12,3 mmHg (p < 0,05) após injeção intravenosa de 10 e 30 µmol/kg de LASSBio-2062 nos SHR. A frequência cardíaca alterou-se de 248,1 ± 12,1 para 146,5 ± 21,0 e de 228,4 ± 11,3 para 109,8 ± 21,4 bpm com doses diferentes de LASSBio-2062 ( Figura 6 ).

## Discussão

A semelhança nas propriedades químicas do enxofre e do selênio incentivou o *design* , a síntese e a avaliação comparativa de uma ampla variedade de moléculas contendo selênio. Porém, existem diferenças nas propriedades físico-químicas entre substâncias que contêm enxofre ou selênio em suas estruturas, que constituem a base para a promoção de efeitos biológicos específicos. Entre as diversas funções biológicas promovidas pelo selênio, tem sido descrita a ação antioxidante.^23^

As novas *N* -acilidrazonas testadas visaram identificar novos agentes anti-hipertensivos que pudessem, além de reduzir a pressão arterial, interferir no estresse oxidativo, que é uma condição comum na hipertensão arterial. A comparação entre os compostos demonstrou aumento da potência do relaxamento vascular independente do endotélio induzido pelo LASSBio-2062, que teve substituição do anel tiofênico pelo anel selenofênico. A *N* -metilação observada no LASSBio-2063 proporcionou não somente o aumento da potência da ação vasodilatadora, mas também a independência da integridade do endotélio vascular, semelhantemente ao observado para o LASSBio-785, que apresenta N-metilação em seu estrutura, mas com átomo de enxofre.^24^ A *N* -metilação parece estar envolvida com o aumento da potência, possivelmente porque o grupo metil, ligado à ligação amida da *N* -acilidrazona, é capaz de induzir alteração na conformação da estrutura molecular da substância. Assim, a avaliação desses novos derivados reafirma que as modificações estruturais introduzidas pelo grupo metil são essenciais tanto para o aumento da eficácia e da potência vasodilatadora quanto para alterações nas vias envolvidas para o efeito vascular.^24^ A abertura do anel benzodioxol, além da substituição do átomo de enxofre pelo selênio (LASSBio-2075) resultou em relaxamento vascular dependente da integridade endotelial, diferentemente do LASSBio-430, que possui um átomo de enxofre em sua estrutura.

Dentre todos os derivados testados, apenas o LASSBio-2084 não apresentou melhora na potência em comparação ao protótipo LASSBio-294, indicando que a para-metoxilação reduziu tanto a potência quanto a eficácia da substância para relaxamento vascular, características revertidas pela presença do átomo de selênio (LASSBio-2076). Os derivados metoxilados na meta-posição contendo átomo de enxofre (LASSBio-2092) ou selênio (LASSBio-2093) não apresentaram alteração significativa da potência.

A substituição do anel tiofênico pelo anel selenofênico proporcionou aumento ou manutenção da potência de relaxamento vascular, que foi mediada pela ativação de receptores presentes no endotélio vascular. Assim, a *N* -metilação proporciona relaxamento vascular diretamente relacionado à ação na musculatura lisa vascular, de forma semelhante ao LASSBio-785, que é N-metilado, com átomo de enxofre.^24^ A ação direta na musculatura lisa vascular produzida pelo LASSBio-2063, LASSBio-430 e LASSBio-2092 pode ser vantajosa devido ao fato de que a disfunção endotelial e o remodelamento vascular que ocorrem na hipertensão arterial podem prejudicar a vasodilatação dependente do endotélio.^25^

**Figura 7:**
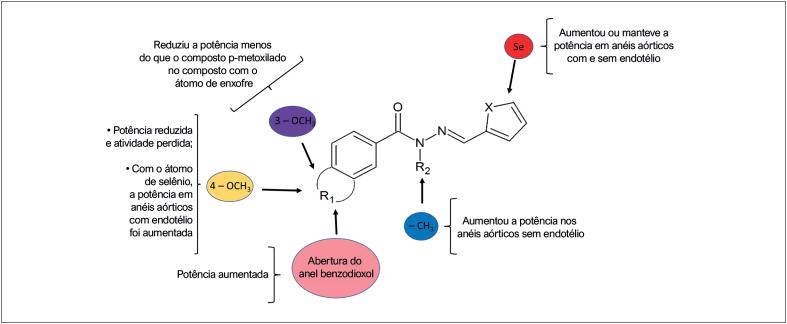
Lista de modificações estruturais e respectivos resultados relacionados à reatividade vascular (Imagem criada por Tadeu L. Montagnoli).

As modificações estruturais e os respectivos resultados principais quanto às alterações de potência dos derivados *N* -acilidrazônicos avaliados neste estudo estão resumidos na Figura 7 .

Os mecanismos envolvidos na ação vasodilatadora e no efeito anti-hipertensivo induzidos pelo LASSBio-2062 foram investigados pelos seguintes motivos: (1) sua semelhança com a estrutura química do LASSBio-294 (protótipo) diferia apenas pela substituição do anel tiofênico pelo anel selenofênico; e (2) apresentou potência 4 vezes maior que o protótipo^26^ em promover relaxamento vascular com ação no endotélio e na musculatura lisa vascular.

A ação vasodilatadora devido à ativação de receptores de adenosina subtipo A_2A_, localizados tanto na musculatura lisa vascular quanto no endotélio, já foi demonstrada anteriormente para muitos derivados *N* -acilidrazônicos.^16 , 26 , 27^ Assim, foi inicialmente investigado o envolvimento de receptores de adenosina A_2A_ para relaxamento vascular induzido pelo LASSBio-2062. A eficácia e a potência não foram alteradas na presença de um antagonista do receptor de adenosina A_2A_, devido ao fato de que a relação entre a concentração e o relaxamento vascular permaneceu inalterada, sugerindo a não participação da ativação desses receptores no tecido vascular ( Figura 3A ).

Visto que a ativação dos receptores de adenosina A_3_ também desempenha um papel importante no processo de relaxamento vascular,^28^ a ação do LASSBio-2062 foi avaliada na presença do antagonista MRE 3008F20. A potência e a resposta máxima de relaxamento vascular foram reduzidas na presença do antagonista do receptor de adenosina A_3_, indicando a participação da ativação desses receptores na vasodilatação induzida por esses derivados ( Figura 3B ). Nas artérias coronárias de ratos, a ativação de receptores de adenosina do subtipo A_3_^29^ pelos agonistas N_6_-(3-iodobenzil)-adenosina-5’-N-metiluronamida (IB-MECA) e 2-cloro-N_6_-(3-iodobenzil)-adenosina-5’-N-metilcarboxamida (Cl-IB-MECA) produz vasodilatação coronária.^30^

**Figura 8:**
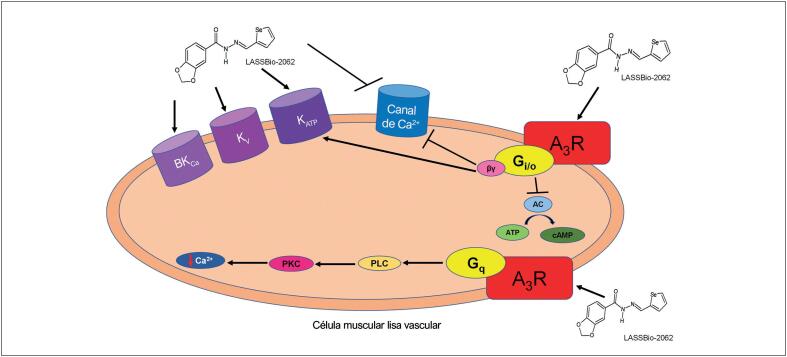
Possíveis mecanismos envolvidos nos efeitos do LASSBio-2062. A ativação do receptor de adenosina A3 pelo LASSBio-2062 pode promover a inibição da AC através da proteína Gi e, consequentemente, levar à redução na produção da AMPc. A subunidade Gβγ pode promover a ativação da PLC, bem como a ativação dos canais de K^+^ e o bloqueio dos canais de Ca^2+^. O receptor de adenosina também está acoplado à proteína Gq, que ativa a PKC e a PLC para promover a vasodilatação. A ativação dos canais K_ATP_ e o bloqueio dos canais de Ca^2+^ tipo L pelo LASSBio-2062 podem ocorrer diretamente neste canal ou como resultado da ativação do receptor de adenosina A_3_. A ativação dos canais de potássio pelo LASSBio-2062 não se restringe ao K_ATP_, pois os antagonistas dos canais K_Ca_ e K_V_ reduziram a vasodilatação. A_3_R: receptor de adenosina subtipo A_3_; AC: adenilato ciclase; AMPc: adenosina monofosfato cíclico; ATP: trifosfato de adenosina; βγ: subunidade βγ; BK_Ca_: canal de potássio cálcio-dependente de alta condutância; Gi/0: proteína Gi/0; Gq: proteína Gq; K : canal de potássio sensível a ATP; K_V_: canal de potássio voltagem-dependente; PKC: proteína quinase C; PLC: fosfolipase C.

A cardioproteção ocorre com a ativação dos receptores de adenosina A_3_, que é abolida pela glibenclamida, um bloqueador do canal de potássio sensível a ATP, indicando que esse receptor pode interferir na ativação desses canais, resultando na sua abertura^31 - 33^ e hiperpolarização celular. Não foi observado relaxamento vascular induzido pelo LASSBio-2062 após pré-incubação com glibenclamida em anéis aórticos sem endotélio funcional ( Figura 4A ). A ativação de canais de potássio sensíveis a ATP expressos na musculatura lisa vascular leva à hiperpolarização da membrana celular do músculo vascular, promovendo vasodilatação.^34^ A ativação dos receptores de adenosina A_3_ influencia a atividade dos canais de K+, especialmente dos canais de potássio sensíveis a ATP, podendo induzir sua abertura,^35 , 36^ o que resulta em hiperpolarização e consequente bloqueio dos canais de Ca^2+^. A redução do influxo de cálcio leva à menor concentração intracelular de cálcio e resulta em vasodilatação^34^ ( Figura 8 ).

Dessa maneira, a ativação do receptor de adenosina A_3_ promove a inibição da adenilato ciclase via proteína Gi e, consequentemente, leva à redução da produção de adenosina monofosfato cíclico. O receptor de adenosina A_3_ também está acoplado à proteína Gq, que ativa a proteína quinase C, que interage com canais de cálcio no retículo sarcoplasmático e canais de potássio sensíveis a ATP para promover vasodilatação.^28 , 31 , 37 , 38^ Além disso, através da ativação de proteínas Gi/o, via subunidades Gβγ, resulta na ativação de canais de potássio sensíveis a ATP e na redução da entrada de cálcio no ambiente intracelular.^39^ A ativação de canais de potássio sensíveis a ATP pelo LASSBio-2062 pode ocorrer diretamente neste canal e/ou através da ativação do receptor de adenosina A_3_. A ativação dos canais de potássio pelo LASSBio-2062 não se restringe ao canal de potássio sensível a ATP, uma vez que a vasodilatação foi inibida pela exposição aos antagonistas dos canais de potássio voltagem-dependentes e cálcio-dependentes ( Figura 4B e 4C , respectivamente).^40 , 41^ A ativação desses canais pode ser um alvo farmacológico alternativo ao tratamento da hipertensão arterial. O maior efluxo de potássio, através de diferentes canais de potássio ativados pelo LASSBio-2062, resultaria em hiperpolarização das células musculares lisas, o que poderia levar ao fechamento dos canais de cálcio e à consequente redução da concentração intracelular de cálcio e vasodilatação. De acordo com os resultados obtidos, o LASSBio-2062 pode promover vasodilatação ativando o receptor de adenosina A_3_ e os canais de K+ e bloqueando diretamente os canais de Ca^2+^, conforme indicado na Figura 8 .

Alterações estruturais dos receptores de adenosina A_3_ ou alterações nos diferentes estágios da via de sinalização interferem no desenvolvimento da hipertensão arterial essencial. Os receptores de adenosina A_3_ encontrados em humanos estão envolvidos em várias funções citoprotetoras, enquanto sua ativação está ligada a efeitos anti-inflamatórios e cardioprotetores.^42^ O agonista do receptor de adenosina A_3_, namodenoson, tem efeitos anti-inflamatórios, antifibróticos e de anti-esteatose, uma vez que mostrou resultados promissores em um estudo de fase III para o tratamento do câncer de fígado e em um estudo de fase II para o tratamento da esteatohepatite não alcoólica. A namodenoson atua ativando os receptores de adenosina A_3_, inibindo a produção de citocinas inflamatórias como fator de necrose tumoral α (TNF-α), interleucina (IL)-12, interferon-ɣ, IL-17 e IL-23 e regulando negativamente o NF-κB.^43^ Existe uma regulação negativa da expressão do receptor de adenosina A_3_ no coração de animais hipertensos,^28^ indicando que esses receptores são potenciais alvos farmacológicos para o tratamento da hipertensão arterial. O efeito vasodilatador do LASSBio-2062 pode ser mediado pela ativação do receptor de adenosina A_3_ e sua administração intravenosa em SHR reduziu a pressão arterial média nas duas doses utilizadas, 10 e 30 μmol/kg (3 e 10 mg/kg). Quando comparamos o efeito anti-hipertensivo do LASSBio-2062 com alguns medicamentos clinicamente disponíveis, identificamos que a inibição dos efeitos da angiotensina II pelo inibidor da enzima conversora de angiotensina enalapril ocorre com a administração intravenosa de 8,2 μg/kg em ratos.^44^ Em contrapartida, a ação anti-hipertensiva desse agonista do receptor de adenosina A_3_ foi comparável à descrição da administração intravenosa em ratos hipertensos do anti-hipertensivo losartana (10 mg/kg de antagonista do receptor da angiotensina II).^45^

A ocorrência de bradicardia após administração intravenosa de LASSBio-2062 pode ser benéfica devido à ausência das características de taquicardia reflexa de muitos medicamentos vasodilatadores.^46^

Devido à ativação dos canais de potássio sensíveis a ATP induzida pelo LASSBio-2062, seria esperado o surgimento de efeitos colaterais adversos, como hiperglicemia, por causa da possível redução da liberação de insulina nas células beta pancreáticas.^41 , 47^ Entretanto, há uma diferença entre as subunidades dos canais de potássio sensíveis a ATP nos vários tecidos onde são expressos, que incluem pâncreas, cérebro, coração e músculo liso e esquelético.^48^ O LASSBio-2062 usado como agente anti-hipertensivo provavelmente não afetaria pacientes com diabetes, pois os canais de potássio sensíveis a ATP presentes nos vasos são diferentes daqueles localizados no pâncreas. A ausência de alteração no nível de glicose plasmática após injeção intravenosa de LASSBio-2062 reforça a falta de interferência nos distúrbios metabólicos. A glicemia foi de 139,5 ± 5,0 e 127,0 ± 11,0 mg/dL 10 e 30 minutos após a injeção intravenosa do veículo. O tratamento com LASSBio-2062 (30 umol/kg, intravenoso) não alterou significativamente esse parâmetro com 172,0 ± 24,0 e149,5 ± 24,0 mg/dL.

O novo agonista do receptor de adenosina A_3_, LASSBio-2062, representa uma alternativa terapêutica para o tratamento da hipertensão arterial, indicando que o sistema de adenosina é um novo potencial alvo farmacológico. A ativação do receptor de adenosina A_3_ tem efeitos benéficos no sistema cardiovascular, pois atenua a condição aterosclerótica,^49^ previne lesão de isquemia/reperfusão miocárdica^50^ e induz vasodilatação coronariana.^28^ Além disso, a ativação desses receptores expressos em neutrófilos, basófilos, eosinófilos e mastócitos reduz a resposta inflamatória.^51 - 54^ Assim, a ativação dos receptores de adenosina A_3_ pode, além de produzir vasodilatação, reduzir o componente inflamatório da hipertensão arterial sistêmica, sugerindo múltiplos efeitos benéficos, que são fatores importantes, por se tratar de uma doença multifatorial. O LASSBio-2062 parece atuar em múltiplos alvos, o que poderia facilitar o uso em regime de monoterapia ou combinado com outros medicamentos, proporcionando interação medicamentosa ideal e reduzindo a dose utilizada. A combinação é capaz de reduzir os efeitos adversos e melhorar o controle da doença.^55^

## Conclusão

Com exceção do LASSBio-2084, todas as *N* -acilidrazonas apresentaram ação vasodilatadora mais potente que o protótipo LASSBio-294, provavelmente devido à substituição do anel tiofênico pelo anel selenofênico. O aumento da potência indica melhora na interação alvo molecular-substância. O relaxamento vascular induzido pelo LASSBio-2062 pode ocorrer através da ativação dos receptores de adenosina A_3_ e da ativação direta/indireta dos canais de potássio. A administração intravenosa de LASSBio-2062 promoveu efeito anti-hipertensivo, sugerindo que o receptor de adenosina A_3_ é um alvo farmacológico inovador para o tratamento da hipertensão arterial.
