# Supplementary material for: Efeito Anti-Hipertensivo de Novos Agonistas do Receptor de Adenosina em Ratos Espontaneamente Hipertensos
Source: Arq Bras Cardiol. 2024 Mar 15;121(2):e20230405. [Article in Portuguese] doi: 10.36660/abc.20230405 (PMC12092035; doi:10.36660/abc.20230405)
Supplement: Translation

# Translation: Antihypertensive Effect of New Agonist of Adenosine Receptor in Spontaneously Hypertensive Rats

**Authors:** Bruna de Souza Rocha, Jaqueline Soares da Silva, Júlia Galvez Bulhões Pedreira, Tadeu Lima Montagnoli, Eliezer Jesus Barreiro, Gisele Zapata-Sudo

**Affiliations:** 1 Rio de Janeiro RJ Brazil Universidade Federal do Rio de Janeiro, Rio de Janeiro, RJ – Brazil

**Keywords:** Hypertension, Vasodilation, Calcium Channels, Purinergic P1 Receptors

## Abstract

**Background:**

Systemic arterial hypertension is a risk factor for cardiac, renal, and metabolic dysfunction. The search for new strategies to prevent and treat cardiovascular diseases led to the synthesis of new N-acylhydrazones to produce antihypertensive effect. Adenosine receptors are an alternative target to reduce blood pressure because of their vasodilatory action and antioxidant properties, which may reduce oxidative stress characteristic of systemic arterial hypertension.

**Objective:**

To evaluate the antihypertensive profile of novel selenium-containing compounds designed to improve their interaction with adenosine receptors.

**Methods:**

Vascular reactivity was evaluated by recording the isometric tension of pre-contracted thoracic aorta of male Wistar rats after exposure to increasing concentrations of each derivative (0.1 to 100 μM). To investigate the antihypertensive effect in spontaneously hypertensive rats, systolic, diastolic, and mean arterial pressure and heart rate were determined after intravenous administration of 10 and 30 μmol/kg of the selected compound LASSBio-2062.

**Results:**

Compounds named LASSBio-2062, LASSBio-2063, LASSBio-2075, LASSBio-2076, LASSBio-2084, LASSBio-430, LASSBio-2092, and LASSBio-2093 promoted vasodilation with mean effective concentrations of 15.5 ± 6.5; 14.6 ± 2.9; 18.7 ± 9.6; 6.7 ± 4.1; > 100; 6.0 ± 3.6; 37.8 ± 11.8; and 15.9 ± 5.7 μM, respectively. LASSBio-2062 (30 μmol/kg) reduced mean arterial pressure in spontaneously hypertensive rats from 124.6 ± 8.6 to 72.0 ± 12.3 mmHg (p < 0.05). Activation of adenosine receptor subtype A_3_and potassium channels seem to be involved in the antihypertensive effect of LASSBio-2062.

**Conclusions:**

The new agonist of adenosine receptor and activator of potassium channels is a potential therapeutic agent to treat systemic arterial hypertension.

## Introduction

According to the World Health Organization, the death of an estimated 23.6 million people will occur by 2030 as a result of cardiovascular diseases.¹ Even with the wide variety of existing treatments, high blood pressure remains the leading cause of cardiac death worldwide, accounting for 10.4 million deaths per year, with the highest number of individuals affected by hypertension in low- and middle-income countries.^2 - 5^

Even though there are several modern therapies for the treatment of hypertension, it remains the main cause of cardiovascular diseases, such as ischemic heart disease, heart failure, and stroke, worsened by population aging.^6 - 9^

Because of the asymptomatic feature, systemic arterial hypertension can progress with structural and/or functional alterations in target organs (heart, brain, kidneys, and vessels).^10^It is important to seek new strategies for the prevention and treatment of hypertension; thus, new *N* -acylhydrazones have been designed, synthetized from the 3,4-methylenedioxybenzoyl-2-thienylhydrazone (LASSBio-294) to test for vasodilatory activity. LASSBio-294, characterized as a positive cardiac inotropic agent with vasodilator activity, prevented cardiac dysfunction induced by myocardial infarction in normotensive and hypertensive rats, possibly due to activation of A_2A_adenosine receptors.^11 - 16^To improve the interaction between the molecule and its site of action, the novel compounds have the sulfur atom (–S) instead of selenium (–Se), which confers a restrict conformation, leading to better compound-receptor interaction^17^and, consequently, increasing the vasodilator potency. Some changes in the molecular structure could generate compounds with multi-target mechanisms of action, favoring the adherence of patients to therapy, since polypharmacy is common for hypertension control in most patients.^18^The search for new therapies for hypertension is an attempt to increase patient adherence to treatment, since this is a major challenge to cardiologists, because of the use of multiple drugs and/or administrations, high incidence of adverse effects and undiagnosed primary disease.^19 , 20^

The main purpose of this study was to identify a new *N* -acylhydrazone, in which the replacement of the sulfur atom by selenium could increase vasodilator activity and produce antihypertensive effect. Based on the results, LASSBio-2062 was selected, which was a potent vasodilator through the activation of adenosine A_3_receptors and K channels. LASSBio-2062 could be a new approach to monotherapy for hypertension since it promoted normalization of blood pressure in spontaneously hypertensive rats (SHR).

## Methods

The experiments were in accordance with the Committee of Ethics on the Use of Animals of the Federal University of Rio de Janeiro (017/19). The animals included in this study were randomly divided, based on weight and/or age. Five male Wistar rats (220 to 250 g) or 4 male SHR (12 to 15 weeks and 220 to 250 g) were used for each protocol conducted in *in vitro* and *in vivo* experiments, respectively. SHR were treated with an intravenous injection of a single dose of LASSBio-2062 and were euthanized with intraperitoneal (IP) administration of thiopental (120 mg/kg) immediately after blood pressure measurement.^21^All animals were kept under temperature control and light/dark cycles of 12 hours, with access to water and food *ad libitum* .

### Compounds

Derivatives ( Figure 1 ) were synthesized and provided by the Laboratory for Evaluation and Synthesis of Bioactive Substances (Laboratório de Avaliação e Síntese de Substâncias Bioativas, LASSBio^®^) of the Federal University of Rio de Janeiro. The prototype, 3,4-methylenedioxybenzoil-2-tienilhydrazone and its analogues were named LASSBio-294, LASSBio-2062, LASSBio-2063, LASSBio-430, LASSBio-2075, LASSBio-2084, LASSBio-2076, LASSBio-2092, and LASSBio-2093.

**Figure 1:** (A) Chemical structure of LASSBio-294. (B) Structure indicating the points of change in the molecule which originated the following analogues: X = S or Se; R_1_= -OCH_2_O- or -3,4-OCH_3_or -3-OCH_3_or -4-OCH_3_and R_2_= -H or -CH_3_.

### In vitro experiments

#### Vascular reactivity

Thoracic aorta from male Wistar rats were removed and cut into 2 to 3 mm rings, which were positioned in experimental chambers containing 20 mL of modified Tyrode solution consisting of the following (in mM): 123 NaCl; 4.7 KCl; 15.5 NaHCO_3_; 1.2 CaCl_2_; 1.2 KH_2_PO_4_; 1.2 MgCl_2_; and 11.5 glucose. They were oxygenated with carbogen mixture at 37 °C and pH 7.4. After 2 hours of equilibrium and under 1 g of initial tension, the aortic rings were initially exposed to 10 μM phenylephrine to promote maximum contraction recorded using a force transducer (MLT884, ADInstruments), connected to an acquisition system (Powerlab, ADInstruments, Australia), using the Lab Chart program (version 7.0, ADInstruments, Australia). Pre-contracted rings were exposed to 10 μM of acetylcholine in order to promote relaxation which, when equal to or greater than 80%, determined the presence of intact endothelium. In contrast, when aortic rings submitted to mechanical removal of the endothelium produced less than 10% of relaxation, there was a lack of functional endothelium. Increasing concentrations (0.1 to 100 μM) of all analogues (LASSBio-2062, LASSBio-2063, LASSBio-430, LASSBio-2075, LASSBio-2084, LASSBio-2076, LASSBio-2092, LASSBio-2093) were added to the chambers containing the phenylephrine-contracted aortic rings to obtain the concentration-relaxation response curves.^22^

We investigated the mechanisms involved in the vascular relaxation induced by the selected compound, LASSBio-2062, with pre-exposure of aortic rings with intact endothelium to an antagonist of adenosine A_2A_receptor, ZM-241385 (0.1 μM). Endothelium-free aortic rings were pre-incubated with one of the following: an adenosine A_3_receptor antagonist (MRE 3008F20, 0.1 μM); an ATP-sensitive potassium channel antagonist (glibenclamide, 10 μM); a voltage-dependent potassium channel blocker (4-aminopyridine, 3 mM); or a high-conductance calcium-dependent potassium channel blocker (tetraethylammonium chloride, 3 mM).

### In vivo experiments

#### Blood pressure measurement

SHR under anesthesia with ketamine (80 mg/kg, IP) and xylazine (15 mg/kg, IP) were submitted to catheterization, using a catheter filled with saline and heparin, connected to a pressure transducer (MLT884, ADInstruments, Australia). This procedure provided the recording of systolic and diastolic blood pressures and heart rate, during intravenous injection of LASSBio-2062 (10 and 30 μmol/kg) through the left jugular vein. Parameters were obtained using an acquisition system (Powerlab, ADInstruments, Australia) and the software Lab Chart (version 7.0, ADInstruments, Australia).

### Statistical analysis

Data are expressed as mean ± standard error of the mean. For each substance tested, we calculated the concentration that caused half-maximal relaxing effect (EC_50_). Two-way ANOVA analysis followed by Tukey's post-test was used to evaluate data from *in vitro* experiments. Analysis by Student's t test was performed on data from *in vivo* experiments. The difference between the experimental groups was considered statistically significant when p < 0.05. Statistical power (β) of 80% and false positive rate (α) of 5% were considered. In order to identify changes with statistical power (β) of 80% and false positive rate (α) of 5%, an experimental number of 5 animals was necessary for the *in vitro* protocol. Thus, the statistical test differentiates responses with a change of at least 15% and an average standard deviation of 8.0%, in percentages relative to the mean of the control group. Regarding *in vivo* protocols, average standard deviation was 7.0%, relative to the control group, with an experimental number of 4 animals for each group. The software http://powerandsamplesize.com/Calculators was used to calculate the sample size.

## Results

### Vascular reactivity in aortic rings with or without intact endothelium

*N* -acylhydrazones were evaluated for the potential to produce vasodilation compared to the prototype (LASSBio-294). Each derivative was tested in aortic rings with and without intact endothelium, which were exposed to increasing concentrations (0.1 to 100 μM). Table 1 includes the EC_50_values in aortic rings. The concentration-vascular relaxation curves for all analogues tested are displayed in Figure 2 . The mechanical removal of endothelium increased the EC_50_from 15.5 ± 6.5 to 49.4 ± 10.0 μM for LASSBio-2062, indicating that its effect is partially dependent on the functional integrity of vascular endothelium ( Figure 2A ). EC_50_values for LASSBio-2063 were 14.6 ± 2.9 and 22.8 ± 8.0 μM in rings with and without endothelium, respectively, suggesting that it is not dependent on endothelial integrity ( Figure 2B ).

**Table 1:** EC_50_of analogues for vascular relaxation in aortic rings from rats

Compound	R_1_	R_2_	X	EC_50_(µM) (with endothelium)	EC_50_(µM) (without endothelium)
LASSBio-294	-OCH_2_O-	H	S	65.4 ± 30.0	> 100*
LASSBio-2062	-OCH_2_O-	H	Se	15.5 ± 6.5	49.4 ± 10.0*
LASSBio-785	-OCH_2_O-	CH_3_	S	10.2 ± 0.5	18.5 ± 3.6
LASSBio-2063	-OCH_2_O-	CH_3_	Se	14.6 ± 2.9	22.8 ± 8.0
LASSBio-430	3,4-OCH_3_	H	S	6.0 ± 3.6	14.1 ± 2.8
LASSBio-2075	3,4-OCH_3_	H	Se	18.7 ± 9.6	> 100*
LASSBio-2084	4-OCH_3_	H	S	> 100	> 100
LASSBio-2076	4-OCH_3_	H	Se	6.7 ± 4.1	60.1 ± 20.2*
LASSBio-2092	3-OCH_3_	H	S	37.8 ± 11.8	59.8 ± 11.9
LASSBio-2093	3-OCH_3_	H	Se	15.9 ± 5.7	> 100*

* *p < 0,05, compared to data with intact endothelium using two-way ANOVA analysis. EC_50_: half-maximal effective concentration; R_1_: radical 1; R_2_: radical 2.*

**Figure 2:** Concentration-vascular response curves for (A) LASSBio-294; (B) LASSBio-2062; (C) LASSBio-2063; (D) LASSBio-2084; (E) LASSBio-2076;(F) LASSBio-430; (G) LASSBio-2075; (H) LASSBio-2092; and (I) LASSBio-2093 in aortic rings with or without functional endothelium. Data are shown as mean ± standard error of the mean. *p < 0.05 compared to intact endothelium. Analysis by two-way ANOVA followed by Tukey's post-test.

The design and synthesis of LASSBio-430 was based on the opening of the 1,3-benzodioxol ring, whose results indicate that this structural change may lead to increased efficacy and potency of vasodilation, with endothelium-independent maximum relaxation of 99.2% ± 0.8% and EC_50_of 6.0 ± 3.6 μM.

In contrast, LASSBio-2075, which has additional presence of selenophene ring in place of the tiophene ring has EC_50_of 18.7 ± 9.6 and the vascular relaxation is dependent on endothelium integrity, because the value became > 100 μM when the endothelium was removed. LASSBio-2084 showed no improvement in potency indicating that methoxylation does not interfere with vascular reactivity. Likewise, LASSBio-2093-induced vascular relaxation was dependent on endothelium integrity since EC_50_increased from 15.9 ± 5.7 to > 100 μM.

The dependence of vascular integrity for the vasodilator action of LASSBio-2076 was confirmed by the increase of EC_50_from 6.7 ± 4.1 to 60.1 ± 20.2 μM. Thus, methoxylation added to the presence of the selenophene ring promoted an increase in the potency of LASSBio-2076 in aortic rings with intact endothelium. The LASSBio-2092 analogue presented EC_50_values of 37.8 ± 11.8 and 59.8 ± 11.9 μM in aortic rings with and without endothelium, respectively.

LASSBio-2062 was selected for the investigation of the mechanisms involved in the vascular relaxation, due to the great structural similarity with the original compound.

### Investigation of the mechanisms involved in the vasodilator action of LASSBio-2062

#### Adenosine receptor pathway

There was no alteration in the vascular relaxation promoted by LASSBio-2062 in the presence of an adenosine A_2A_receptor antagonist, ZM-241385 ( Figure 3A ). However, when the aortic rings were pre-incubated with the adenosine A_3_receptor antagonist, MRE 3008F20, the concentration-response curve showed a shift to the right (Figure 3B). Additionally, the maximum relaxation induced by LASSBio-2062 altered from 72.1% ± 4.0% to 23.6% ± 7.7% in the presence of MRE 3008F20. These data indicate that the compound promotes vasodilation by activation of the adenosine receptor type A_3_, but not adenosine receptor type A_2A_.

**Figure 3:** Concentration-vascular relaxation curves of LASSBio-2062 in aortic rings with endothelium: (A) in the absence or presence of ZM-241385 (0.1 μM), an adenosine A_2A_receptor antagonist; and (B) in the presence of MRE 3008F20 (0.1 μM), an adenosine A_3_receptor antagonist. Data are shown as mean ± standard error of the mean. *p < 0.05 compared to intact endothelium. Analysis by two-way ANOVA followed by Tukey's post-test.

#### Involvement of potassium channels

When aortic rings were pre-incubated with glibenclamide (10 μM), which is an ATP-sensitive potassium channel blocker, the response of increasing concentrations of LASSBio-2062 was altered ( Figure 4A ). When in the presence of 4-aminopyridine ( Figure 4B ) or tetraethylammonium chloride (Figure 4C), the concentration-relaxation curve for LASSio-2062 shifted to the right, indicating that LASSBio-2062-induced vasodilation can be consequent to the activation of ATP-sensitive potassium, voltage-dependent potassium, and calcium-dependent potassium channels ( Figure 4 ).

**Figure 4:** Concentration-vascular relaxation curves of LASSBio-2062 in aortic rings without endothelium in the absence or presence of: (A) glibenclamide (10 μM), an ATP-sensitive potassium channel blocker; (B) 4-aminopyridine (4-AP, 3 mM), a voltage-dependent potassium channel blocker; and (C) tetraethylammonium chloride (TEA, 3 mM), a high-conductance calcium-dependent potassium channel blocker. Data are expressed as mean ± standard error of the mean. *p < 0.05 compared to control. Analysis by two-way ANOVA followed by Tukey's post-test.

#### Influence of calcium influx on the action of *N* -acylhydrazones

In order to verify whether LASSBio-2062 induced vasodilation through an additional interference on intracellular Ca^2+^concentration, endothelium-free aortic rings were exposed to increasing concentrations of CaCl_2_(1 to 1000 µM), in the absence or presence of analogue (50 μM) ( Figure 5 ). The concentration-response curve was observed to shift to the right with exposure to LASSBio-2062 with an increase of EC_50_from 157.6 ± 51.3 to 420.0 ± 11.0 μM (p < 0.05).

**Figure 5:** Concentration-response curves of CaCl_2_in the absence or presence of LASSBio-2062 in aortic rings without endothelium. Data are expressed as mean ± standard error of the mean. *p < 0.05 compared to control. Analysis by two-way ANOVA followed by Tukey's post-test

#### Antihypertensive effect of LASSBio-2062

After intravenous administration of LASSBio-2062, systolic pressure of SHR was reduced from 155.1 ± 10.1 to 110.6 ± 8.0 and from 151.1 ± 10.0 to 109.4 ± 13.8 mmHg at a dose of 10 and 30 μmol/kg, respectively. Diastolic pressure was also reduced by administration of LASSBio-2062 from 111.0 ± 9.1 to 63.9 ± 14.7 (10 μmol/kg) and from 100.2 ± 7.8 to 54.0 ± 10.4 mmHg (30 μmol/kg). Mean arterial pressure decreased from 132.0 ± 9.2 and 124.6 ± 8.6 mmHg (control group) to 82.1 ± 12.6 (p < 0.05) and 72.0 ± 12.3 mmHg (p < 0.05) after intravenous injection of 10 and 30 µmol/kg of LASSBio-2062 in the SHR. Heart rate altered from 248.1 ± 12.1 to 146.5 ± 21.0 and from 228.4 ± 11.3 to 109.8 ± 21.4 bpm with different doses of LASSBio-2062 ( Figure 6 ).

**Figure 6:** Hemodynamic parameters observed in spontaneously hypertensive rats before (control) and after intravenous administration of LASSBio-2062.(A) Systolic pressure; (B) diastolic pressure; (C) mean pressure; and (D) heart rate, measured before and after administration 10 μmol/kg (n = 4) and 30 μmol/kg (n = 4) of LASSBio-2062. Data are expressed as mean ± standard error of the mean. *p < 0.05 versus control. Analysis by Student's t test.

## Discussion

The similarity in the chemical properties of sulfur and selenium has encouraged the design, synthesis, and comparative evaluation of a wide variety of selenium-containing molecules. However, there are differences in the physicochemical properties between substances that contain sulfur or selenium in their structures, which constitute the basis for the promotion of specific biological effects. Among the various biological functions promoted by selenium, antioxidant action has been described.^23^

Novel *N* -acylhydrazones tested aimed to identify new antihypertensive agents, which could, in addition to reducing blood pressure, interfere with oxidative stress, a common condition in arterial hypertension. The comparison among compounds demonstrated an increase of potency of the endothelium-independent vascular relaxation induced by LASSBio-2062, which had a replacement of the thiophene ring by selenophene. The *N* -methylation observed in LASSBio-2063 provided not only the increase in the potency of the vasodilator action, but also independence on the integrity of the vascular endothelium, similarly to that observed for LASSBio-785, which presents *N* -methylation in its structure, but with a sulfur atom.^24^ *N* -methylation seems to be involved with the increase in potency, possibly because the methyl group, linked to the amide bond of *N* -acylhydrazone, could induce change in the conformation of the molecular structure of the substance. Thus, the evaluation of these new derivatives reaffirms that the structural modifications introduced by the methyl group are essential for both the increase in efficacy and vasodilator potency and changes in the pathways involved for vascular effect.^24^The opening of the benzodioxol ring, added with the replacement of the sulfur atom by selenium (LASSBio-2075) resulted in vascular relaxation dependent on endothelial integrity, unlike LASSBio-430, which has a sulfur atom in its structure.

Among all tested derivatives, only LASSBio-2084 showed no improvement in potency compared to the prototype LASSBio-294, indicating that para-methoxylation reduced both the potency and efficacy of the substance for vascular relaxation, characteristics reversed by the presence of the selenium atom (LASSBio-2076). Methoxylated derivatives at the meta-position containing a sulfur atom (LASSBio-2092) or selenium (LASSBio-2093) did not show significant potency alteration.

Replacement of the thiophene ring by the selenophene ring provided increase or maintenance of the potency of vascular relaxation, which was mediated by activation of receptors present in the vascular endothelium. Thus, *N* -methylation provides vascular relaxation directly related to the action on vascular smooth muscle, similarly to LASSBio-785, which is *N* -methylated, with a sulfur atom.^24^The direct action on vascular smooth muscle produced by LASSBio-2063, LASSBio-430 and LASSBio-2092 may be advantageous due to the fact that endothelial dysfunction and vascular remodeling which occur in arterial hypertension could impair endothelium-dependent vasodilation.^25^

The structural modifications and the respective main results regarding the changes in potency of the *N* -acylhydrazonic derivatives evaluated in this work are summarized in Figure 7 .

**Figure 7:** List of structural modifications and respective results related to vascular reactivity (Image created by Tadeu L. Montagnoli).

Mechanisms involved in the vasodilator action and antihypertensive effect induced by LASSBio-2062 were investigated because of the following: (1) its similarity to the chemical structure of LASSBio-294 (prototype) differing only by the replacement of the thiophene ring by selenophene; and (2) the fact that it showed potency 4 times greater than the prototype^26^in promoting vascular relaxation with action on the endothelium and vascular smooth muscle.

Vasodilator action due to the activation of adenosine receptors subtype A_2A_located both in the vascular smooth muscle and in the endothelium has previously demonstrated for many *N* -acylhydrazonic derivatives.^16,26,27^Thus, it was initially investigated the involvement of adenosine A_2A_receptors for vascular relaxation induced by LASSBio-2062. The efficacy and potency were not altered in the presence of an adenosine A_2A_receptor antagonist, due to the fact that the concentration-vascular relaxation relationship remained unchanged, suggesting the non-participation of activation of these receptors in the vascular tissue (Figure 3A).

Since the activation of adenosine A_3_receptors also plays an important role in the process of vascular relaxation,^28^the action of LASSBio-2062 was evaluated in the presence of the antagonist MRE 3008F20. The maximum vascular relaxing response and potency were reduced in the presence of the A_3_adenosine receptor antagonist, indicating the participation of the activation of these receptors in the vasodilation induced by these derivatives (Figure 3B). In rat coronary arteries, activation of adenosine receptors of subtype A_3_^29^by agonists N_6_-(3-iodobenzyl)-adenosine-5’-N-methyluronamide (IB-MECA) and 2-chloro-N_6_-(3-iodobenzyl)-adenosine-5’-N-methylcarboxamide (Cl-IB-MECA) produces coronary vasodilation.^30^

Cardioprotection occurs with activation of adenosine A_3_receptors, which is abolished by glibenclamide, an ATP-sensitive potassium channel blocker, indicating that this receptor may interfere with the activation of these channels, resulting in their opening^31 - 33^and cellular hyperpolarization. No LASSBio-2062-induced vascular relaxation was observed after pre-incubation with glibenclamide on aortic rings without functional endothelium (Figure 4A). The activation of ATP-sensitive potassium channels expressed in vascular smooth muscle leads to hyperpolarization of the vascular muscle cell membrane, promoting vasodilation.^34^The activation of adenosine A_3_receptors influences the activity of K^+^channels, especially ATP-sensitive potassium channels, and may induce their opening,^35 , 36^which results in hyperpolarization and consequent blockade of Ca^2+^channels. The reduced influx of calcium leads to lower intracellular calcium concentration and results in vasodilation^34^( Figure 8 ).

**Figure 8:** Possible mechanisms involved in the effects of LASSBio-2062. The activation of the A_3_adenosine receptor by LASSBio-2062 could promote inhibition of AC through Gi protein and, consequently, lead to a reduction in cAMP production. Gβγ subunit, could promote PLC activation, as well as activation of K^+^channels and blockade of Ca^2+^channels. The adenosine receptor is also coupled to the Gq protein, which activates PKC and PLC to promote vasodilation. Activation of K_ATP_channels and blockade of membrane L-type Ca^2+^channels by LASSBio-2062 could occur directly on this channel or as result of activation of the A_3_adenosine receptor. Activation of potassium channels by LASSBio-2062 is not restricted to K_ATP_, because K_Ca_and K_v_channel antagonists reduced vasodilation. A_3_R: adenosine receptor subtype A_3_; AC: adenylate cyclase; ATP: adenosine triphosphate; βγ: subunit βγ; BK_Ca:_high conductance calcium-dependent potassium channel; cAMP: cyclic adenosine monophosphate; Gi/0: Gi/0 protein; Gq: Gq protein; K_ATP_: ATP-sensitive potassium channel; K_v_: voltage-gated potassium channel; PKC: protein kinase C; PLC: phospholipase C.

Thus, the activation of the adenosine A_3_receptor promotes the inhibition of adenylate cyclase via Gi protein, and, consequently, leads to a reduction in cAMP production. Adenosine A_3_receptor is also coupled to the Gq protein, which activates protein kinase C, which interacts with calcium channels in the sarcoplasmic reticulum and ATP-sensitive potassium channels to promote vasodilation.^28,31,37,38^Furthermore, through the activation of Gi/o proteins, via Gβγ subunits, it results in the activation of ATP-sensitive potassium channels and the reduction of calcium entry into the intracellular environment.^39^Activation of ATP-sensitive potassium channels by LASSBio-2062 could occur directly in this channel and/or through the activation of adenosine A_3_receptor. The activation of potassium channels by LASSBio-2062 is not restricted to ATP-sensitive potassium channel, since vasodilation was inhibited by exposure to voltage-dependent potassium and calcium-dependent potassium channel antagonists (Figure 4B e 4C, respectively).^40 , 41^Activation of these channels can be an alternative pharmacological target to the treatment of arterial hypertension. The greater efflux of potassium, through different K channels activated by LASSBio-2062, would result in hyperpolarization of smooth muscle cells, which could lead to the closure of calcium channels and consequent reduction of intracellular calcium concentration and vasodilation. According to the results obtained, LASSBio-2062 could promote vasodilation by activating adenosine A_3_receptor and K^+^channels and directly blocking Ca^2+^channels, as indicated in Figure 8 .

Structural alterations of adenosine A_3_receptors or changes in the different stages of the signaling pathway interfere with the development of essential arterial hypertension. Adenosine A_3_receptors found in humans are involved in several cytoprotective functions while its activation is linked to anti-inflammatory and cardioprotective effects.^42^The adenosine A_3_receptor agonist namodenoson has anti-inflammatory, antifibrotic, and anti-steatosis effects, since it has shown promising results in a phase III study for the treatment of liver cancer and in a phase II study for the treatment of nonalcoholic steatohepatitis. Namodenoson acts by activating adenosine A_3_receptors, inhibiting the production of inflammatory cytokines such as tumor necrosis factor α (TNF-α), interleukin (IL)-12, interferon-ɣ, IL-17 and IL-23 and negatively regulating NF-κB.^43^There is a negative regulation of adenosine A_3_receptor expression in the hearts of hypertensive animals,^28^indicating that those receptors are potential pharmacological targets for treatment of arterial hypertension. The vasodilatory effect of LASSBio-2062 could be mediated by activation of the adenosine A_3_receptor and its intravenous administration in SHR reduced mean arterial pressure at both doses used, 10 and 30 μmol/kg (3 and 10 mg/kg). When we compared the antihypertensive effect of LASSBio-2062 with some clinically available drugs, we identified that the inhibition of the effects of angiotensin II by the angiotensin-converting enzyme inhibitor enalapril occurs with intravenous administration of 8.2 μg/kg in rats.^44^In contrast, the antihypertensive action of this adenosine A_3_receptor agonist was comparable to the description that intravenous administration in hypertension rats of the antihypertensive losartan (10 mg/kg angiotensin II receptor antagonist).^45^

The occurrence of bradycardia after intravenous administration of LASSBio-2062 could be beneficial because of the absence of the reflex tachycardia characteristics of many vasodilator drugs.^46^

Due to the activation of ATP-sensitive potassium channels induced by LASSBio-2062, the advent of adverse side effects, such as hyperglycemia, would be expected, because of possible reduction of insulin release in pancreatic beta cells.^41 , 47^However, there is a difference between the subunits of ATP-sensitive potassium channels in the various tissues where they are expressed, which include the pancreas, brain, heart, and smooth and skeletal muscle.^48^LASSBio-2062 used as an antihypertensive agent probably would not affect patients with diabetes, because the ATP-sensitive potassium channels present in the vessels are different from those located in the pancreas. Absence of change in plasmatic glucose level after intravenous injection of LASSBio-2062 reinforces the lack of interference in metabolic disorders. Blood glucose was 139.5 ± 5.0 and 127.0 ± 11.0 mg/dL 10 and 30 minutes after intravenous injection of vehicle. Treatment with LASSBio-2062 (30 umol/kg, intravenous) did not significantly alter this parameter with 172.0 ± 24.0 and 149.5 ± 24.0 mg/dL.

The new agonist of adenosine A_3_receptor, LASSBio-2062, represents a therapeutic alternative for the treatment of arterial hypertension, indicating that the adenosine system is a new potential pharmacological target. Activation of adenosine A3 receptor has beneficial effects on the cardiovascular system, since it attenuates atherosclerotic condition,^49^prevents myocardial ischemia-reperfusion injury,^50^and induces coronary vasodilation.^28^Additionally, the activation of those receptors expressed in neutrophils, basophils, eosinophils, and mast cells reduces the inflammatory response.^51 - 54^Thus, activation of adenosine A3 receptors could, in addition to producing vasodilation, reduce the inflammatory component of systemic arterial hypertension, suggesting multiple beneficial effects, which are important factors, since it is a multifactorial disease. LASSBio-2062 seems to act on multiple targets, which could facilitate the use in monotherapy regimen or combined with other drugs, providing ideal drug interaction and reducing the dose used. The combination could reduce adverse effects and improve disease control.^55^

## Conclusion

Except for LASSBio-2084, all N-acyhydrazones showed more potent vasodilator action than the prototype LASSBio-294, probably because of the replacement of the thiophene ring by selenophene. Increased potency indicates improvement in the molecular target-substance interaction. LASSBio-2062-induced vascular relaxation may occur through the activation of A_3_adenosine receptors and direct/indirect activation of K channels. Intravenous administration of LASSBio-2062 promoted antihypertensive effect, suggesting that the adenosine A_3_receptor is an innovative pharmacological target for the treatment of arterial hypertension.
